# Robotic Cancer Surgery

**DOI:** 10.3390/cancers13194931

**Published:** 2021-09-30

**Authors:** Jens Hoeppner

**Affiliations:** Department of Surgery, University Medical Center of Schleswig-Holstein-Campus Lübeck, Ratzeburger Allee 160, 23538 Lübeck, Germany; jens.hoeppner@uksh.de

Cancer kills millions of people around the world every year. Surgery is a mainstay for the cure of solid organ cancer. As a modern principle of surgical oncology, surgery should not only remove the complete locoregional tumor, but also aim to minimize surgical trauma to the patient in order to reduce postoperative morbidity and to improve and guarantee postoperative quality of life.

Compared to open surgery, minimally invasive surgical techniques can provide benefits in the form of reduced blood loss, faster recovery, less pain, less wound-related complications and a reduced hospital stay for the patient. Therefore, minimally invasive surgery techniques with instrumentation by a video camera and special surgical instruments, which are inserted through small incisions in the patient’s skin, are used in surgical oncology.

Robotic surgery is one of the most recent innovations in the spectrum of minimally invasive surgery. After introduction of the robotic platform for surgical procedures in the beginning of the current millennium, the first operations were conducted for a variety of benign diseases. Nowadays, robotic surgery is also increasingly applied in complex cancer surgery. Robotic-assisted surgical techniques, especially for tumors at solid organs located in visceral cavities, were developed, approved and distributed within the last decade.

Current technology in the field of robotic surgery can improve surgical outcomes in treating cancer through improved and highly magnified 3DHD-visualization and intra operative near-infrared fluorescence imaging with visual assessment of tumor tissue and related tissue perfusion. Complex anatomical areas can be visualized through high-resolution and three-dimensional (3D) vision with high magnification options and a very stable operating field. The preparation under this view can separate tissue structures from one another very precisely and, thus, minimize intraoperative injuries to critical surrounding anatomical structures.

Besides the improved visualization, high-precision instrument control and movement is a major improvement obtained by robotic instrumentation. Robotic instruments allow endo-wristed movements and improve surgical dexterity and expand the options for minimally invasive tumor resectability and surgical reconstruction. The scaling of motion in the movement of robotic instruments can improve the precision and safety of surgical procedures. All of these factors contribute to minimizing surgical trauma, increasing the precision and safety of the tumor resection and increasing the safety of reconstruction procedures. This improves the functional but, in some cases, also the oncological results of tumor operations.

Today, robotic operations are performed on tumor diseases of the lung, prostate, esophagus, pancreas, kidney, intestine, cervix and other organs. In particular, the feasibility and safety of the application of surgical robots have been largely proven “in field” for various cancer entities and surgical approaches. However, surgical, technical and scientific challenges remain. Clear long-term evidence of the superior results of curative robotic surgery over traditional open and especially conventional laparoscopic approaches is controversial.

Despite all these experiences and possibilities, the use of robotic systems in surgical oncology must always be critically questioned. In particular, the question of whether real evidence exists for these postulated advantages must be asked. The definitively more cost-intensive robotic method can only be economically justified, compared to the traditional approaches, with proof of its superiority for the relevant functional and oncologic endpoints. 

Obtaining this evidence will be one of the greatest challenges in surgical oncology over the next few years. Up-to-date, available, prospective randomized therapy comparison trials are very rare and mostly show no or only marginal advantages for the use of robotic systems in surgical oncology. Corresponding individual studies are available, e.g., for rectal cancer and bladder cancer [1,2]. Many surgeons already work with the support of surgical robots in their everyday life and in the routine care of their patients. Nevertheless, less high-level evidence is rare compared to the international distribution and everyday application of complex surgical robots. Neglecting to generate this evidence would hinder the further development of robotic cancer surgery in the medium and long term and most likely result in disadvantages for future patients.

This special edition of *Cancers* shows the current results of robotic cancer surgery and broadens the evidence for the application of the technique in surgical oncology. It also discusses the limits and shows future possibilities for improving the surgical results of cancer treatment through the use of surgical robots.
